# Crystal structure of methyl 2-[5-(2-hy­droxy­phen­yl)-2*H*-tetra­zol-2-yl]acetate

**DOI:** 10.1107/S205698901701698X

**Published:** 2017-11-30

**Authors:** Seul Gi Lee, Ji Yeon Ryu, Junseong Lee

**Affiliations:** a Department of Chemistry, Chonnam National University, Gwangju, 61186, Republic of Korea

**Keywords:** crystal structure, tetra­zole, hy­droxy­phenyl tetra­zole, hydrogen bonding, offset π–π inter­actions

## Abstract

The title compound, C_10_H_10_N_4_O_3_, was synthesized by the esterification of hy­droxy­phenyl tetra­zole. There is an intra­molecular O—H⋯N hydrogen bond present involving the hy­droxy group and the tetra­zole ring. The tetra­zole ring is inclined to the phenol ring by 2.85 (13)°, while the methyl acetate group is almost normal to the tetra­zole ring, making a dihedral angle of 82.61 (14)°. In the crystal, mol­ecules are linked by pairs of C—H⋯O hydrogen bonds, forming inversion dimers. Within the dimers, the phenol rings are linked by offset π–π inter­actions [inter­centroid distance = 3.759 (2) Å]. There are no further significant inter­molecular inter­actions present in the crystal. The hy­droxy group is disordered about positions 2 and 6 on the benzene ring, with a refined occupancy ratio of 0.531 (5):0.469 (5).

## Abstract 2

The title compound, methyl 2-[5-(2-hy­droxy­phen­yl)-2*H*-tetra­zol-2-yl]acetate, is the major product from the reaction between 2-(2*H*-tetra­zol-5-yl)phenol and methyl 2-bromo­acetate in the presence of potassium carbonate, which gave three isomeric products.

## Chemical context   

Tetra­zole ligands are useful building blocks for the construction of high-dimensional metal–organic frameworks by providing various binding modes toward metal centers (Karaghiosoff *et al.*, 2009 ▸; Liu *et al.*, 2013 ▸). Recently, we have used 5-(2-hy­droxy­phen­yl)tetra­zole as a chelating multidentate ligand and reported several inter­esting compounds (Park *et al.*, 2015 ▸; 2014 ▸). It provides strong [N,O] chelation to metal centers with various additional binding modes. As part of a project on the study of the substitution effects on the tetra­zole ring on the self-assembly behaviour in solution, as well as in the solid state, we have synthesized a number of substituted hy­droxy­phenyl tetra­zole complexes. The substitution of the tetra­zole group may promote supra­molecular inter­action by weak inter­actions, such as hydrogen bonding. The reaction between hy­droxy­phenyl tetra­zole and bromo acetate methyl ester in the presence of potassium carbonate gave three isomeric products. Using column chromatography, the major product was isolated and its mol­ecular structure was determined unambiguously by X-ray crystallography. We report herein, the synthesis and crystal structure of this compound.
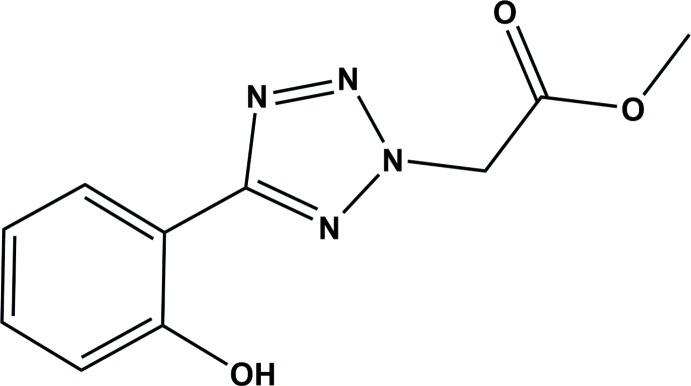


## Structural commentary   

**Figure 1:**
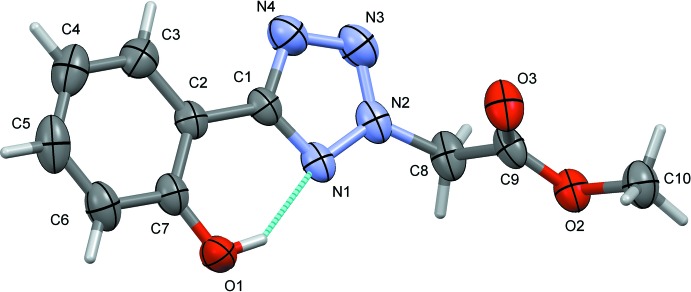
A view of the mol­ecular structure of the title compound, with the atom labelling and 30% probability displacement ellipsoids. The intra­molecular O—H⋯N hydrogen bond (see Table 1 ▸) is indicated by a dashed line. Only the major component of the disordered OH group, in position 2, is shown.

The mol­ecular structure of the title compound is shown in Fig. 1 ▸. The structure analysis confirms the nature of the major product of the reaction, which yielded three isomeric compounds as described in Section 5, *Synthesis and crystallization*. The title mol­ecule consists of a tetra­zole ring (N1–N4/C1) and a phenol ring (C2–C7), which are connected by an intra­molecular O—H⋯N hydrogen bond (Fig. 1 ▸, Table 1 ▸) and inclined to one another by 2.85 (13)°. The planar methyl acetate group [O2/O3/C8–C10; maximum deviation of 0.037 (2) Å for atom O2] is inclined to the tetra­zole ring by 82.61 (14)°.

## Supra­molecular features   

**Figure 2:**
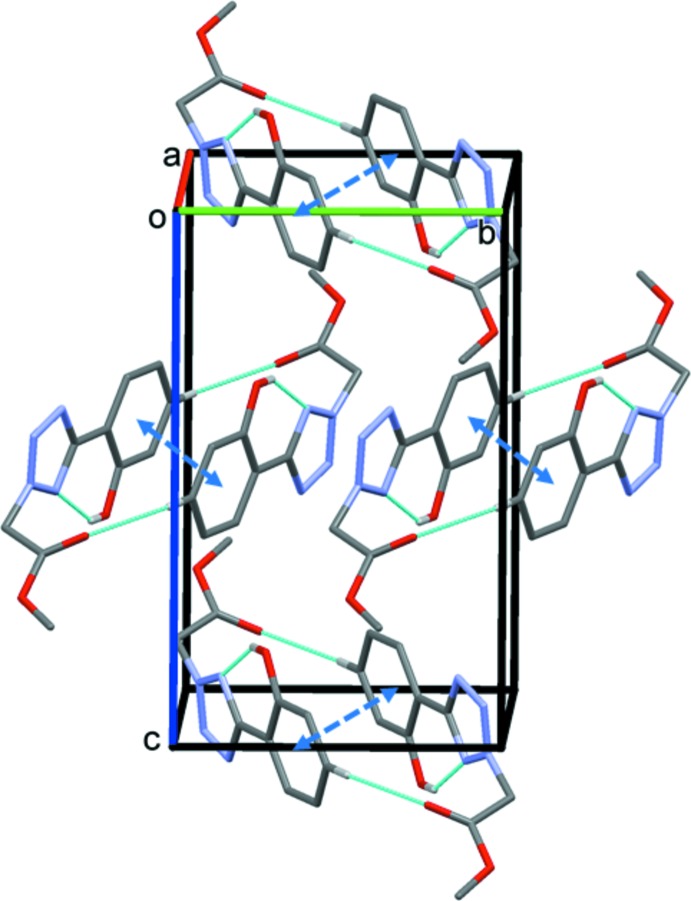
A view along the *a* axis of the crystal packing of the title compound. The intra- and inter­molecular hydrogen bonds (see Table 1 ▸) are indicated by dashed lines. The offset π–π inter­actions are shown as dashed double arrows. Only H atoms H1 and H5, and the major component of the disordered OH group in position 2, have been included.

In the crystal, the mol­ecules are linked by pairs of C—H⋯O hydrogen bonds, forming inversion dimers with an 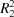
(22) loop (Table 1 ▸, Fig. 2 ▸). Within the dimers, the phenol rings are linked by offset π–π inter­actions [*Cg*⋯*Cg* ^i^ = 3.759 (2) Å, inter­planar distance = 3.526 (1) Å, slippage 1.305 Å; *Cg* is the centroid of the C2–C7 phenol ring, symmetry code: (i) −*x* + 1, −*y*, −*z* + 1]. There are no further significant inter­molecular inter­actions present in the crystal.

## Database survey   

A search of the Cambridge Structural Database (Version 5.38, update May 2017; Groom *et al.*, 2016 ▸) for the methyl 2-(5-phenyl-2*H*-tetra­zol-2-yl)acetate skeleton revealed only two hits, *viz*. ethyl (*Z*)-3-phenyl-2-(5-phenyl-2*H*-tetra­zol-2-yl)-2-propenoate (SAKVIM; Ramazani *et al.*, 2017 ▸) and methyl (5-phenyl-2*H*-tetra­zol-2-yl)acetate (WUKNUN; Saeed *et al.*, 2015 ▸). In WUKNUN, the 5-phenyl substituent is inclined to the tetra­zole ring by 3.89 (7)°, compared to 2.85 (13)° in the title compound. In contrast, the corresponding dihedral angle in SAKVIM is 19.97 (16)°. The meth­yl/ethyl acetate groups are inclined to the plane of the tetra­zole ring by 84.99 (7)° in WUKNUN and 84.57 (7)° in SAKVIM, similar to the value observed in the title compound, *viz*. 82.61 (14)°.

## Synthesis and crystallization   

**Figure 3:**
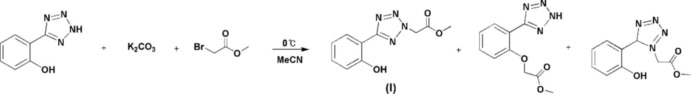
Reaction scheme for the synthesis of the title compound, (I).

The synthesis of the title compound is illustrated in Fig. 3 ▸. 2-(2*H*-Tetra­zol-5-yl)phenol (100 mg, 0.62 mmol) and potassium carbonate (85.0 mg, 0.62 mmol) were dissolved in aceto­nitrile at 273 K while stirring for 30 min. To the resulting solution methyl 2-bromo­acetate (207 µl, 2.18 mmol) was added and stirring was continued for 24 h. The white solid that was obtained was filtered and the solvent removed under reduced pressure. The residue was purified by column chromatography on silica gel using ether:hexane (2:3) as eluent. Three isomeric compounds were obtained, as shown in Fig. 3 ▸. The major product (I) (yield = 59%), was recrystallized in di­chloro­methane and yielded needle-like colourless crystals of the title compound. Spectroscopic data: ^1^H NMR (CDCl_3_, 400MHz): *δ* = 9.59 (*s*, 1H, OH), 8.06 (*d*, 1H, Ph), 7.41 (*t*, 1H, Ph), 7.11 (*d*, 1H, Ph), 6.99 (*t*, 1H, Ph), 5.51 (*s*, 2H), 3.85 (*s*, 3H). ^13^C NMR (125 MHz, CDCl_3_): 165.06, 164.68, 156.42, 132.44, 127.50, 120.06, 117.62, 53.41, 53.38 ppm.

## Refinement   

Crystal data, data collection and structure refinement details are summarized in Table 2 ▸. The hy­droxy group is disordered about positions 2 and 6 on the phenol ring, with a refined occupancy ratio of 0.531 (5):0.469 (5). All the H atoms were included in calculated positions using a riding model: O—H = 0.84 Å, C-H = 0.95–1.00 Å with *U*_iso_(H) = 1.5 *U*_eq_(O-hydroxyl, C-meth­yl) and 1.2*U*_eq_(C) for other H atoms.

## Supplementary Material

Crystal structure: contains datablock(s) I, Global. DOI: 10.1107/S205698901701698X/su5409sup1.cif

Structure factors: contains datablock(s) I. DOI: 10.1107/S205698901701698X/su5409Isup2.hkl

Supporting information file. DOI: 10.1107/S205698901701698X/su5409Isup3.cml

CCDC reference: 1587621

Additional supporting information:  crystallographic information; 3D view; checkCIF report

## Figures and Tables

**Table 1:** Hydrogen-bond geometry (Å, °)

*D*—H⋯*A*	*D*—H	H⋯*A*	*D*⋯*A*	*D*—H⋯*A*
O1—H1⋯N1	0.84	1.91	2.659 (4)	148
C5—H5⋯O3^i^	0.95	2.57	3.472 (3)	158

Symmetry code: (i) 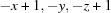.

**Table 2:** Experimental details

Crystal data	
Chemical formula	C_10_H_10_N_4_O_3_
*M*_r_	234.22
Crystal system, space group	Monoclinic, *P*2_1_/*c*
Temperature (K)	100
*a*, *b*, *c* (Å)	10.060 (2), 8.2538 (17), 13.536 (3)
β (°)	104.479 (10)
*V* (Å^3^)	1088.2 (4)
*Z*	4
Radiation type	Mo *K*α
μ (mm^−1^)	0.11
Crystal size (mm)	0.15 × 0.10 × 0.10

Data collection	
Diffractometer	Bruker APEXII CCD
Absorption correction	Multi-scan (*SADABS*; Krause *et al.*, 2015 ▸)
*T*_min_, *T*_max_	0.987, 0.989
No. of measured, independent and observed [*I* > 2σ(*I*)] reflections	14003, 2372, 1252
*R*_int_	0.044
(sin θ/λ)_max_ (Å^−1^)	0.642

Refinement	
*R*[*F* ^2^ > 2σ(*F* ^2^)], *wR*(*F* ^2^), *S*	0.057, 0.137, 1.02
No. of reflections	2372
No. of parameters	167
H-atom treatment	H-atom parameters constrained
Δρ_max_, Δρ_min_ (e Å^−3^)	0.14, −0.17

Computer programs: *APEX2* and *SAINT* (Bruker, 2014 ▸), *SHELXS97* (Sheldrick, 2008 ▸), *SHELXL2014* (Sheldrick, 2015 ▸), *Mercury* (Macrae *et al.*, 2008 ▸) and *publCIF* (Westrip, 2010 ▸).

## supplementary crystallographic information

### Crystal data

**Table:**

C_10_H_10_N_4_O_3_	*F*(000) = 488
*M**_r_* = 234.22	*D*_x_ = 1.430 Mg m^−^^3^
Monoclinic, *P*2_1_/*c*	Mo *K*α radiation, λ = 0.71073 Å
*a* = 10.060 (2) Å	Cell parameters from 3134 reflections
*b* = 8.2538 (17) Å	θ = 2.9–24.3°
*c* = 13.536 (3) Å	µ = 0.11 mm^−^^1^
β = 104.479 (10)°	*T* = 100 K
*V* = 1088.2 (4) Å^3^	Needle, colourless
*Z* = 4	0.15 × 0.10 × 0.10 mm

### Data collection

**Table:**

Bruker APEXII CCD diffractometer	1252 reflections with *I* > 2σ(*I*)
φ and ω scans	*R*_int_ = 0.044
Absorption correction: multi-scan (SADABS; Krause *et al.*, 2015)	θ_max_ = 27.2°, θ_min_ = 2.1°
*T*_min_ = 0.987, *T*_max_ = 0.989	*h* = −12→12
14003 measured reflections	*k* = −10→10
2372 independent reflections	*l* = −17→17

### Refinement

**Table:**

Refinement on *F*^2^	Primary atom site location: structure-invariant direct methods
Least-squares matrix: full	Secondary atom site location: difference Fourier map
*R*[*F*^2^ > 2σ(*F*^2^)] = 0.057	Hydrogen site location: inferred from neighbouring sites
*wR*(*F*^2^) = 0.137	H-atom parameters constrained
*S* = 1.01	*w* = 1/[σ^2^(*F*_o_^2^) + (0.0479*P*)^2^ + 0.3171*P*] where *P* = (*F*_o_^2^ + 2*F*_c_^2^)/3
2372 reflections	(Δ/σ)_max_ < 0.001
167 parameters	Δρ_max_ = 0.14 e Å^−^^3^
0 restraints	Δρ_min_ = −0.17 e Å^−^^3^

### Special details

**Table:**

Geometry. All esds (except the esd in the dihedral angle between two l.s. planes) are estimated using the full covariance matrix. The cell esds are taken into account individually in the estimation of esds in distances, angles and torsion angles; correlations between esds in cell parameters are only used when they are defined by crystal symmetry. An approximate (isotropic) treatment of cell esds is used for estimating esds involving l.s. planes.

### Fractional atomic coordinates and isotropic or equivalent isotropic displacement parameters (Å^2^)

**Table:**

	*x*	*y*	*z*	*U*_iso_*/*U*_eq_	Occ. (<1)
O1	0.3781 (3)	0.2337 (4)	0.3644 (3)	0.0745 (14)	0.531 (5)
H1	0.4534	0.2755	0.3623	0.112*	0.531 (5)
O2	0.92819 (16)	0.46762 (19)	0.26859 (14)	0.0718 (5)	
O3	0.92563 (18)	0.2756 (2)	0.38419 (16)	0.0879 (6)	
O1A	0.5939 (5)	0.1877 (6)	0.7094 (3)	0.1031 (19)	0.469 (5)
H1A	0.6557	0.2377	0.6900	0.155*	0.469 (5)
N1	0.61929 (19)	0.3786 (2)	0.43476 (16)	0.0618 (5)	
N2	0.7415 (2)	0.4500 (2)	0.46016 (19)	0.0679 (6)	
N3	0.8043 (2)	0.4384 (3)	0.5571 (2)	0.0890 (7)	
N4	0.7203 (2)	0.3567 (3)	0.59958 (17)	0.0826 (7)	
C1	0.6085 (2)	0.3209 (3)	0.5240 (2)	0.0574 (6)	
C2	0.4922 (2)	0.2276 (2)	0.53816 (19)	0.0555 (6)	
C3	0.4899 (3)	0.1677 (3)	0.6336 (3)	0.0749 (7)	
H3	0.5638	0.1911	0.6908	0.090*	0.531 (5)
C4	0.3811 (4)	0.0744 (3)	0.6462 (3)	0.0916 (10)	
H4	0.3810	0.0334	0.7117	0.110*	
C5	0.2743 (3)	0.0412 (3)	0.5647 (3)	0.0913 (10)	
H5	0.2001	−0.0237	0.5734	0.110*	
C6	0.2733 (3)	0.1010 (3)	0.4701 (3)	0.0831 (8)	
H6	0.1978	0.0790	0.4137	0.100*	
C7	0.3818 (3)	0.1929 (3)	0.4565 (2)	0.0652 (7)	
H7	0.3808	0.2330	0.3905	0.078*	0.469 (5)
C8	0.8045 (3)	0.5246 (3)	0.3866 (2)	0.0778 (8)	
H8A	0.8619	0.6170	0.4189	0.093*	
H8B	0.7322	0.5670	0.3288	0.093*	
C9	0.8923 (2)	0.4048 (3)	0.3474 (2)	0.0635 (7)	
C10	1.0223 (3)	0.3749 (3)	0.2257 (2)	0.0834 (8)	
H10A	1.0402	0.4334	0.1674	0.125*	
H10B	0.9817	0.2691	0.2031	0.125*	
H10C	1.1087	0.3594	0.2776	0.125*	

### Atomic displacement parameters (Å^2^)

**Table:**

	*U*^11^	*U*^22^	*U*^33^	*U*^12^	*U*^13^	*U*^23^
O1	0.063 (2)	0.086 (3)	0.071 (3)	−0.0122 (18)	0.0098 (17)	0.0043 (19)
O2	0.0666 (11)	0.0501 (10)	0.1035 (13)	0.0043 (8)	0.0303 (10)	0.0086 (10)
O3	0.0893 (13)	0.0446 (10)	0.1425 (17)	0.0147 (9)	0.0529 (12)	0.0194 (11)
O1A	0.127 (4)	0.118 (4)	0.062 (3)	0.006 (3)	0.018 (3)	0.011 (3)
N1	0.0533 (12)	0.0445 (11)	0.0897 (15)	0.0028 (10)	0.0215 (11)	−0.0002 (11)
N2	0.0570 (13)	0.0463 (12)	0.1035 (18)	0.0007 (10)	0.0260 (13)	−0.0040 (12)
N3	0.0684 (15)	0.0838 (18)	0.111 (2)	−0.0131 (14)	0.0151 (15)	−0.0115 (16)
N4	0.0699 (15)	0.0835 (16)	0.0897 (17)	−0.0084 (13)	0.0113 (13)	−0.0090 (13)
C1	0.0534 (15)	0.0414 (12)	0.0779 (17)	0.0082 (11)	0.0172 (13)	−0.0068 (13)
C2	0.0568 (14)	0.0374 (12)	0.0754 (17)	0.0095 (11)	0.0225 (13)	−0.0017 (12)
C3	0.085 (2)	0.0605 (17)	0.082 (2)	0.0139 (15)	0.0271 (18)	0.0054 (16)
C4	0.118 (3)	0.0586 (18)	0.119 (3)	0.0109 (19)	0.069 (2)	0.0116 (18)
C5	0.096 (2)	0.0504 (17)	0.151 (3)	−0.0090 (16)	0.074 (2)	−0.015 (2)
C6	0.0686 (18)	0.0668 (18)	0.121 (3)	−0.0077 (15)	0.0376 (17)	−0.0194 (18)
C7	0.0604 (16)	0.0517 (15)	0.088 (2)	0.0008 (12)	0.0273 (15)	−0.0047 (14)
C8	0.0703 (16)	0.0433 (14)	0.130 (2)	0.0011 (12)	0.0439 (17)	0.0049 (15)
C9	0.0474 (13)	0.0384 (13)	0.106 (2)	−0.0044 (11)	0.0219 (13)	−0.0004 (14)
C10	0.0779 (18)	0.0708 (18)	0.110 (2)	0.0042 (15)	0.0398 (16)	−0.0052 (16)

### Geometric parameters (Å, º)

**Table:**

O1—C7	1.282 (4)	N4—C1	1.351 (3)
O2—C9	1.315 (3)	C1—C2	1.452 (3)
O2—C10	1.447 (3)	C2—C7	1.387 (3)
O3—C9	1.190 (3)	C2—C3	1.388 (3)
O1A—C3	1.280 (5)	C3—C4	1.383 (4)
N1—C1	1.328 (3)	C4—C5	1.362 (4)
N1—N2	1.329 (3)	C5—C6	1.370 (4)
N2—N3	1.310 (3)	C6—C7	1.378 (3)
N2—C8	1.445 (3)	C8—C9	1.508 (3)
N3—N4	1.319 (3)		
			
C9—O2—C10	117.19 (18)	O1A—C3—C4	119.1 (4)
C1—N1—N2	101.8 (2)	O1A—C3—C2	119.9 (3)
N3—N2—N1	114.2 (2)	C4—C3—C2	120.7 (3)
N3—N2—C8	122.4 (2)	C5—C4—C3	120.0 (3)
N1—N2—C8	123.2 (2)	C4—C5—C6	120.3 (3)
N2—N3—N4	105.9 (2)	C5—C6—C7	120.1 (3)
N3—N4—C1	106.5 (2)	O1—C7—C6	116.2 (3)
N1—C1—N4	111.6 (2)	O1—C7—C2	122.9 (3)
N1—C1—C2	124.2 (2)	C6—C7—C2	120.7 (3)
N4—C1—C2	124.2 (2)	N2—C8—C9	111.09 (19)
C7—C2—C3	118.1 (2)	O3—C9—O2	126.0 (2)
C7—C2—C1	121.0 (2)	O3—C9—C8	124.7 (2)
C3—C2—C1	120.8 (2)	O2—C9—C8	109.3 (2)

### Hydrogen-bond geometry (Å, º)

**Table:**

*D*—H···*A*	*D*—H	H···*A*	*D*···*A*	*D*—H···*A*
O1—H1···N1	0.84	1.91	2.659 (4)	148
C5—H5···O3^i^	0.95	2.57	3.472 (3)	158

Symmetry code: (i) −*x*+1, −*y*, −*z*+1.
